# Unique and shared partner priorities for supporting engagement in knowledge mobilization in pediatric pain: a best–worst scaling experiment

**DOI:** 10.1186/s12961-025-01310-2

**Published:** 2025-04-18

**Authors:** Nicole E. MacKenzie, Christine T. Chambers, Deborah A. Marshall, Christine E. Cassidy, Penny V. Corkum, Meghan E. McGrady, Jennifer A. Parker, Karen V. MacDonald, Kathryn A. Birnie

**Affiliations:** 1 Department of Psychology and Neuroscience, Dalhousie University, Life Sciences Centre, 1355 Oxford St, Halifax, NS B3H 4J1 Canada; 2 Centre for Pediatric Pain Research, IWK Health, Halifax, NS Canada; 3 Department of Pediatrics, Dalhousie University, Halifax, NS Canada; 4 Department of Community Health Sciences, Cumming School of Medicine, University of Calgary, Calgary, AB Canada; 5 School of Nursing, Dalhousie University, Halifax, NS Canada; 6 IWK Health, Halifax, NS Canada; 7 Department of Psychiatry, Dalhousie University, Halifax, NS Canada; 8 Department of Pediatrics, IWK Health, Halifax, NS Canada; 9 Division of Behavioral Medicine and Clinical Psychology, Cincinnati Children’s Hospital Medical Center, Cincinnati, OH United States of America; 10 Department of Pediatrics, University of Cincinnati College of Medicine, Cincinnati, OH United States of America; 11 Department of Anesthesiology, Perioperative, and Pain Medicine, Cumming School of Medicine, University of Calgary, Calgary, AB Canada

**Keywords:** Partnership, Knowledge mobilization, Best–worst scaling, Choice experiment, paediatric pain

## Abstract

**Background:**

Engaging in partnerships is key to the success of knowledge mobilization (KM) activities; however, how best to engage partners in KM activities in the context of paediatric pain and children’s health more broadly is not well understood. There is limited guidance on what supports the development of effective partnerships in KM activities with a variety of partner types. The purpose of this study was to examine the preferences and priorities of three partner groups (i.e. health professionals, researchers and patient/caregiver partners) when it comes to supporting their engagement in KM activities within paediatric pain and children’s health.

**Methods:**

We used a case 1 (object case) best–worst scaling (BWS) experiment, a stated preferences method to assess priorities and relative importance of factors related to supporting engagement in KM activities and compare their importance across the three partner groups. Participants completed 12 tasks requiring them to select items that were most and least important to supporting their engagement in KM activities. A total of 11 items, generated through a previous elicitation task, were included in the balanced incomplete block experimental design for the BWS. Difference scores and ratio values were calculated for each group and relative comparisons were observed across groups.

**Results:**

A total of 127 participants completed the BWS experiment. All partner groups agreed that items related to relationships within teams were among the most important, while pragmatic items related to executing KM were amongst the least important. While there was relative similarity in the items ranked as important, varying priorities also emerged for each group; fit of KM activities in the clinical context was particularly important among researchers, while flexible communication was relatively more important within the patient/caregiver group. Health professionals differed the least from the other groups.

**Conclusions:**

Different partner groups prioritized strong relationships when it comes to supporting engagement in KM activities, reinforcing the importance of connections in KM processes. There was nuance, however, around how partner groups valued various aspects of relationships. Individuals leading KM initiatives in paediatric pain and children’s health should discuss relationships and pragmatics with partners to ensure successful collaboration and impactful activities.

**Supplementary Information:**

The online version contains supplementary material available at 10.1186/s12961-025-01310-2.

## Introduction

The effective management of paediatric pain (i.e. pain of any cause or duration in children from birth to age 18 years) is paramount not only in management of primary pain conditions (e.g. complex regional pain syndrome), but also acute, procedural and chronic pain management in the context of other childhood illness and disease. Despite the availability of evidence-based practices to manage pain, knowledge of evidence and implementation remains a primary barrier to its use [1]. Knowledge mobilization (KM; i.e. dissemination and implementation of evidence in clinical practice and policy) is essential to ensure that awareness of, and access to, evidence is facilitated among those who may benefit from it, such as health professionals, researchers, as well as patients and their caregivers [2]. KM is facilitated through the “push” of evidence from researchers to those who are impacted by it, through the “pull” of information by those who require evidence, and the “exchange” of knowledge among those who produceevidence and those who use it [3]. KM activities have increased within paediatric pain, through initiatives such as Solutions for Kids in Pain (SKIP), a national KM network dedicated to leveraging relationships to mobilize knowledge about paediatric pain and its management [4].

Partnership is a fundamental component of effective and impactful KM. Partnership in KM involves meaningfully engaging relevant and interested parties, or partners, in developing the approach with which evidence will be mobilized [5]. In healthcare contexts, partners often include the researchers who co-produce evidence, health professionals who work within the clinical settings where evidence will be mobilized, and patients and their caregivers who may co-produce evidence, provide context on its use in practice and use it themselves [6]. When partners are engaged in KM, dissemination and implementation activities have greater relevance to the intended knowledge user audience, have greater clinical impact and are more feasible to adopt into practice [7–9].

Effective partnerships in knowledge co-production have the potential to improve capacity to share evidence, improve quality of initiatives and increase value of research [8, 10]. Facilitators of partnership approaches include open communication, willingness to collaborate, time, experience engaging partners, remuneration and resources [10–12], while differences in attitudes and priorities, as well as power dynamics that exist between different partners on teams, have been identified as barriers to engaging in KM [8, 11]. Solutions to these barriers include establishing partnerships with clarity around roles, leveraging partner expertise and supporting partners in engaging within the partnership [11, 13]. Understanding these factors may provide a foundation for informing an approach to partnerships in KM activities; however, there are important gaps that exist. First, the majority of the available literature, models and frameworks on partnership in KM have focused on knowledge co-production (i.e. research) [14], a distinct activity from KM. Moreover, a recent evidence synthesis indicated that partners are inconsistently engaged in KM activities arising from research [15], suggesting that not only is little known about how to engage in partnership for KM specifically, but also that it is practised inconsistently. Indeed, there is a general gap in literature highlighting how to engage in partnership specifically for the purpose of KM activities [6, 16, 17]. Thus, specific evidence to support partnership within KM is necessary, as end-of-grant KM or clinical implementation activities may occur without a preceding co-produced research project. This is especially important in the context of paediatric pain, where partners have specifically identified the need for greater support to engage in KM activities [1]. Moreover, partners are often youth with pain, and their caregivers represent a patient partner group that is unique relative to other settings with primarily adult patient partners.

The available evidence on factors promoting integrated partnership approaches may provide a foundational understanding of how partnerships in research and knowledge co-production are approached; however, how different partners engage in KM activities is not well understood. Indeed, managing and reconciling discrepancies in priorities (i.e. factors or values that are important to individuals) within partnerships themselves has been identified as a challenge within partnerships [8]. A more detailed and structured understanding of how to effectively engage different partner types in KM activities, especially within the area of paediatric pain, is necessary. Previous work by this group of researchers [18] explored what different types of partners considered important to facilitate their engagement in KM activities within children’s pain; however, no research has examined the priorities of these groups when it comes to how they are engaged and participate in KM activities, nor have these priorities been compared. This understanding is essential to identify common and unique considerations to inform how best to engage in partnerships for KM activities with a range of partners, with the ultimate goal of effective implementation to support children’s pain management practices across medical settings.

The purpose of this study was to examine the priorities and preferences of health professionals, researchers and patient/caregiver partners related to what they believe is most important to supporting their engagement in KM activities within paediatric pain and pain-related areas of children’s health.

## Methods

### Study design

The use of experimental methods in implementation science offers the potential for novel contributions to this area, specifically by facilitating a better understanding of factors and mechanisms underlying behaviour [19]. This study utilized a case 1 (object case) best–worst scaling (BWS) experiment within a cross-sectional survey to examine individuals’ preferences and priorities from a predetermined set of items (i.e. factors of interest) [20]. BWS experiments have been widely used in the healthcare and health economics literature to answer a range of questions pertaining to the provision of, and encounters with, healthcare (e.g. value of healthcare outcomes, experience factors and preferences for treatment) [21], as well as implementation in healthcare [22, 23], with a variety of respondents (e.g. patients, health professionals and knowledge producers) [24]. The present study used case 1 (object case) BWS, which facilitates the examination of the importance of items in a choice set relative to each other by indicating the items that are most and least important to an individual in a given scenario [25]. To design the object case BWS, a series of steps was taken, including item generation, the balanced incomplete block design experimental design generation, and survey pretesting and pilot testing. The study design and results were reported in line with the Strengthening the Reporting of Observational Studies in Epidemiology (STROBE) guidelines (Additional File 1).

#### Item generation

**Fig. 1:**
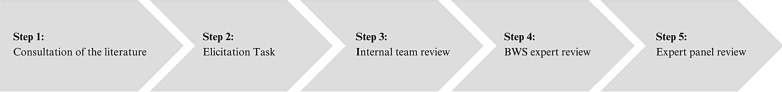
Item generation process

In line with best practices for BWS [25, 26], items were developed with three primary recommendations: consultation of literature to generate attributes, an elicitation task and review of the items (see Fig. 1). First, the KM literature was consulted to familiarize the authors with the barriers and facilitators to KM activities, as well as relevant implementation frameworks. After consulting the literature, the Consolidated Framework for Implementation Research (CFIR) was selected as the guiding framework to inform the preliminary items of interest, given its broad inclusion of factors that impact implementation, especially in contexts where implementation has not been well studied [27]. Relevant characteristics were extracted from the CFIR domains, as were relevant barriers and facilitators that aligned with those identified by a previous needs assessment on KM in paediatric pain [1]. This work informed the previously completed elicitation task, administered to 30 participants from three partner groups (i.e. health professionals, researchers and patient/caregiver partners) via semi-structured interview (see [18]). These results generated themes describing factors of relevance to each partner group to support their engagement in KM activities within paediatric pain (see [18] for complete results). A preliminary list of items was generated from these results, considering relevance to the context, coherence, practicality, independence from other items, nondominance (i.e. not so important that no other items mattered) and nonsubordinance (i.e. not so unimportant that it would never be selected) [26, 28]. To ensure the relevance, clarity and completeness of the proposed items, the items then underwent three rounds of iterative review, including internal study team review, review by an expert in BWS and preferences methods (DAM) and review by an expert panel (i.e. two representatives from the health professional, researcher and patient/caregiver partner groups). In each round, reviewers provided feedback on the basis of the criteria above, and items were removed or revised, resulting in 11 items (see Table 1 for items and definitions).

**Table 1:** BWS item list and definitions

Item	Definition
A collaborative leadership style	Leadership that takes all team member perspectives and opinions into consideration; structures discussions so that all team members can contribute and participate in decision-making
A culture of openness and respect for team members’ perspectives and contributions	A sense of safety, trust, and respect within team member relationships where individuals can openly and freely share their perspectives and experiences
A flexible implementation plan	A plan for carrying out the KM activity that provides a general approach to the KM activity but can also be changed and adjusted as needed (e.g. timeline, tasks, etc.)
Access to a network (e.g. patient/caregiver partner, professional, etc.)	Ability to access and utilize a network of individuals with relevant interests, professional backgrounds, expertise, lived experience, etc.
Fit of the KM activity within the context where the activity will be shared/applied	Fit refers to the relevance and appropriateness of the fit of the KM activity within the context it will be shared/applied in. Context considerations can include how well the KM activity aligns staff needs, clinic workflow, demands on staff to participate in or utilize the KM activity, etc.
Flexible communication methods within teams	Options for a range of methods to engage in communication and meetings, such as virtual meetings, email updates, brief one-on-one updates as needed, etc.
Having a shared goal and commitment to the KM activity among team members	Team members all work towards a common and clear KM goal
Having access to resources to support engagement in KM activities	Availability and ability to access and use resources to make carrying out KM initiatives feasible. Resources can include time, funding, training, personnel, etc.
Having team members with various types of expertise	Teams that consist of individuals with different professional backgrounds and lived experiences interacting with the healthcare system
Personal knowledge of how to lead or participate in KM processes	Personally having knowledge about KM processes. Knowledge may include experience engaging in KM processes, possessing KM-related skills, knowledge of relevant theories/frameworks and other components related to carrying out or participating in a KM initiative
Presence of a “champion” on a KM team	An individual who promotes the importance of KM and empowers and motivates team members to engage in a KM initiative

#### Experimental design

This study used a balanced incomplete block design (BIBD), designed following Louviere et al.’s catalogue [29] and generated using R (R version 4.2.2). The BIBD indicates the number of items and choice tasks to be included within the experiment. The design for 11 items included 11 choice tasks (i.e. questions) with five or six items in each. Study pretesting was conducted to determine whether five or six items would appear in each choice task (see BWS Pretesting Procedure section below). The final design included 11 items, 11 choice tasks with 5 items per choice task, and had a rho (item balance, first order balance) of five and lambda (item co-balance, second order balance) of two [29].

#### BWS pretesting procedure

Survey pretesting is a best practice in choice experiments to ensure the survey complexity is appropriate for the sample population, to identify issues with survey completion, to ensure feasible length and to ensure that questions and instructions are clear [25, 26]. The first author conducted survey pretesting through cognitive interviews via video conferencing (i.e. Zoom). The six experts (i.e. health professionals, researchers and patient/caregiver partners) who reviewed the items also reviewed the survey and provided feedback on the clarity of the BWS task explanation, clarity of the individual BWS questions and instructions, appropriateness of survey length for their partner type and appropriateness of the number of items in each choice set (i.e. question). As a result of this feedback, question phrasing was revised and it was determined that five items would appear in each choice set.

### Participants

**Fig. 2:**
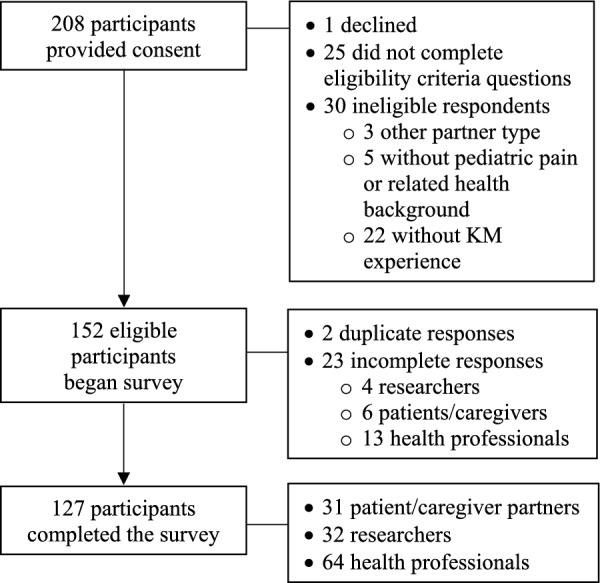
Recruitment flowchart

Eligible participants included any health professionals (e.g. psychologists, physicians, physiotherapists and nurses), researchers (i.e. trainee to senior career) and patient/caregiver partners (18 years of age or older). All participants required experience engaging in at least one KM activity (e.g. resource development, advisory committee participation or clinical practice change activities) within the field of paediatric pain or in an area of children’s health involving pain (e.g. pain as a symptom of illness or disease, procedure pain). Participants who did not have experience with KM activities within paediatric pain or a chronic health condition with pain as a component, as well as participants 17 years of age or younger, were ineligible to participate. Participants that did not complete all choice sets were considered to have incomplete responses. Participants were recruited using convenience and snowball sampling via social media, a research participant database within the Chambers’ research lab, listserv emails, partner organizations, research programs, chronic pain clinic physician lists, webpages and newsletters. Recruitment began August 2023 and ended February 2024. Of the 61 organizations contacted, 67.2% (*n* = 41) agreed to share the recruitment material within their organization networks, while 32.8% (*n* = 20) did not reply, and 0.01% (*n* = 1) declined to share. Of the 90 paediatric pain clinics contacted via the Pain in Childhood special interest group of the International Association for the Study of Pain, 18.9% (*n* = 17) agreed to share within their clinic teams, 65.6% (*n* = 59) did not reply and 15.6% (*n* = 14) of emails were undeliverable. Participation was open to Canadian and international English-speaking participants. While there are no specific guidelines presently available to inform minimum sample sizes required to analyse a BWS experiment [20], past experiments have been conducted with sample sizes as small as 15 participants [24, 25]. Other ways the appropriateness of the sample size was monitored included selecting an efficient experimental model with the fewest number of choice sets to optimize sample size and reviewing responses to ensure there was variability in responding (i.e. that all items had been selected at least once). A total of 208 participants consented to participate in the survey (see Fig. 2). A total of 152 eligible participants began the survey, and 127 participants completed the survey in its entirety—64 health professionals, 32 researchers and 31 patient/caregiver partners. Using a chi-squared test, it was determined that there were no significant differences between partner groups in terms of the proportion of participants who did not complete the survey (*χ*^2^(2) = 0.70, *p* = 0.72).

#### Fraudulent responses

Fraudulent and illegitimate responses, predominantly generated by artificial intelligence or so-called "bots," were received. The online survey utilized reCAPTCHA to automatically screen for bots. All responses were screened for legitimacy by checking for responses from identical internet protocol (IP) addresses and screening survey responses for illogical and/or inconsistent responses and short response times (i.e. less than 10 minutes). Screening was led by one member of the research staff and double-screened by the first author. A total of 486 responses were deemed to be fraudulent and were removed from the dataset.

### Measures

**Fig. 3:**
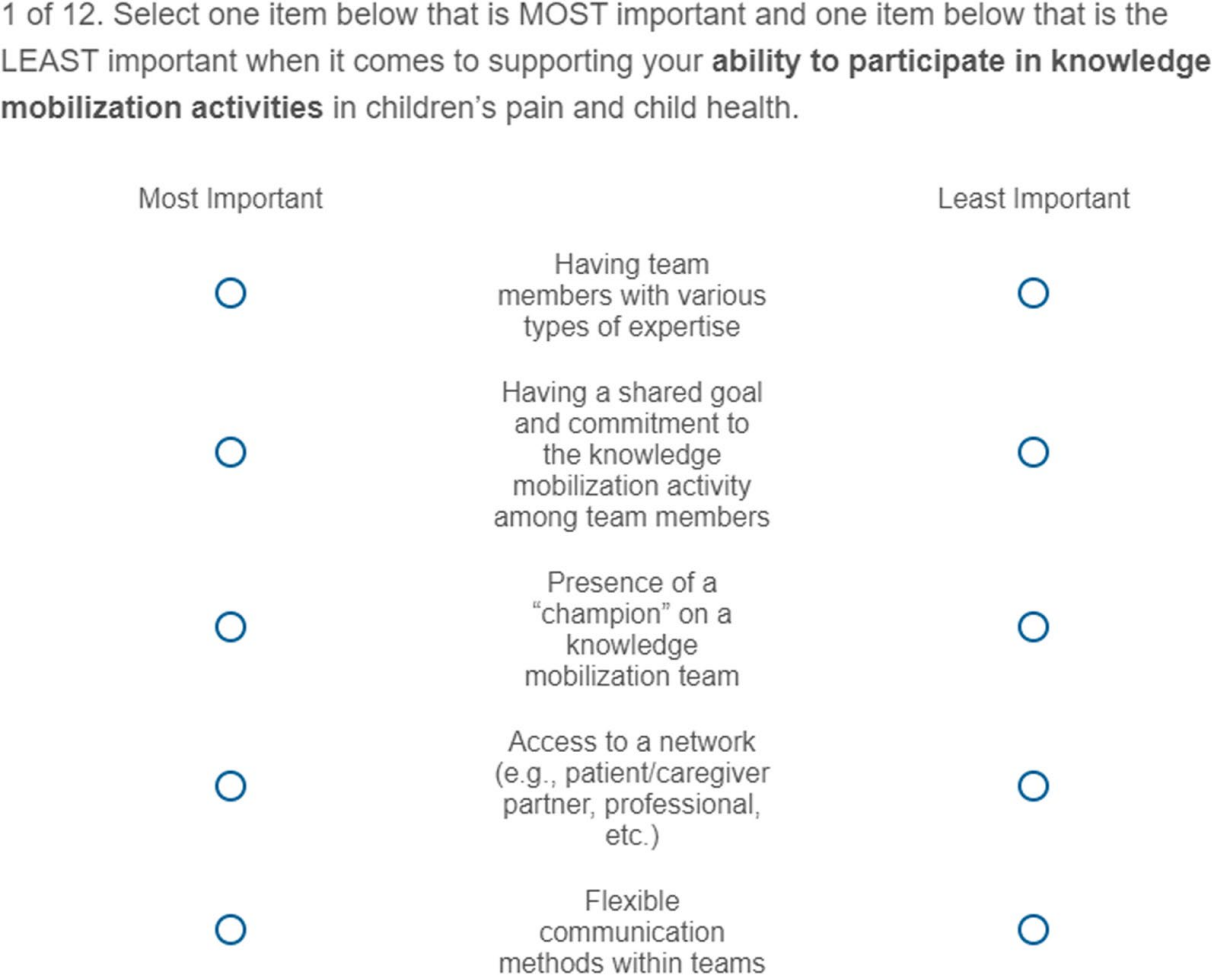
Example choice set

The BWS online survey began with 13 self-report background questions to determine eligibility and characterise participants’ areas of expertise, areas of experience related to paediatric pain, type of KM experience, and expertise in KM. An explanation of how to complete the BWS questions with an example was provided next. The design for 11 items indicated 11 choice tasks (i.e. questions) with five or six items in each. In the survey, 12 BWS choice tasks with five items in each were presented (see Fig. 3), with one choice set as a duplicate question to assess response consistency. Participants reviewed each choice set and selected the items that were most and least important to them when it came to supporting their participation in KM activities within paediatric pain and health more broadly. Data quality was then checked via two methods to ensure validity of responses, in line with the broader preferences methods literature [30–34]. First, participants were asked about whether they understood the choice questions (i.e. yes, no, unsure) and then were asked how frequently they considered all items when responding (i.e. every time, more than half the time, less than half the time, never). Finally, the survey concluded with six demographic questions (e.g. age, education, ethnicity).

### Procedure

This study was approved by the IWK Health Research Ethics Board (REB no. 1027459). Participants reviewed a detailed consent form prior to beginning the survey. Participants reviewed definitions of terminology used within the survey, including KM, implementation and dissemination. Survey responses were collected via Qualtrics, an online survey platform [35]. All participants who completed the survey had the opportunity to enter a draw to receive 1 of 12 $25 (Canadian dollars) online gift cards. Participants also had the opportunity to opt into future research and to receive study results when available.

### Data analysis

Only complete responses (i.e. all BWS questions answered) were included in the analysis owing to the analytic approach. Background characteristics, response quality and demographic variables were summarised using descriptive statistics and frequencies. BWS data were analysed using two approaches: count analysis and ratio scores.

For the count analysis, the frequency with which each item was selected as most and least important was calculated and the difference of these values generated a so-called difference score, which provides an indication of the importance of each item relative to the others. Item rankings within each group were generated on the basis of difference scores, where higher positive difference scores indicate greater importance of an item.

Ratio values were used to interpret the magnitude of difference in importance between items. For the ratio scores, the frequency with which each item was selected as most important was divided by the frequency with which each corresponding item was selected as least important. Then, the square root of each value was taken to generate a ratio value on a pseudo-ratio scale [29, 36]. The natural logs of the ratio values were taken to centre the values around zero [29]. The ratio value standardises the values such that aggregated data can be more reliably interpreted across groups [25, 37, 38]. Each ratio value was then rescaled between 0 to 1 for ease of interpretation, where 0 represented least important and 1 represented most important. Rankings were compared between partner groups on the basis of the rescaled values.

Data quality was checked in two ways to ensure integrity of responses. First, frequencies were generated for response quality questionnaire items (e.g. did you consider each item every time). Second, the responses received for the duplicate choice tasks (i.e. questions) were compared within each participant to determine the extent to which responses changed between the two choice sets (i.e. did participants provide identical responses across both).

## Results

### Participant characteristics

A total of 127 participants completed the survey, a sample comprising 64 participants who identified primarily as health professionals, 32 as researchers and 31 as patient/caregiver partners. Overall, participants predominantly identified as white, cisgender women with a doctorate degree (see Table 2 for demographics). Participants also provided information regarding their professional backgrounds and experiences engaging in KM activities within children’s pain and health more broadly (see Table 3 for all partner characteristics). Most health professionals described their level of experience with engaging in KM as “expert”, and had an average of 15.61 years of experience engaging in KM activities (range = 1–40, standard deviation (SD) = 10.39). Researchers predominantly described their experience with KM as “advanced”, and had an average of 8.28 years of experience engaging in KM activities (range = 2–40, SD = 7.17). Most patient/caregiver partners reported their level of experience engaging in KM activities as “competent” and had an average of 6.48 years of experience engaging in KM activities (range = 1–30, SD = 6.43).

**Table 2:** Participant demographics

	Partner group		
	Health professionals *n* (%)	Researchers *n* (%)	Patient/caregiver partners *n* (%)
Gender			
Cisgender woman	52 (81.30)	27 (84.40)	28 (90.30)
Woman (prefer not to specify)	2 (3.10)	5 (15.60)	1 (3.20)
Cisgender man	7 (10.90)	0 (0.00)	1 (3.20)
Another gender	0 (0.00)	0 (0.00)	1 (3.20)
Prefer not to answer	3 (4.70)	0 (0.00)	0 (0.00)
Race			
Black	0 (0.00)	1 (3.10)	2 (6.50)
East Asian	1 (1.60)	0 (0.00)	1 (3.20)
Latin American	1 (1.60)	0 (0.00)	0 (0.00)
Middle Eastern	1 (1.60)	1 (3.10)	1 (3.20)
South Asian	3 (4.70)	1 (3.10)	3 (9.70)
Southeast Asian	1 (1.60)	1 (3.10)	1 (3.20)
White	55 (85.90)	28 (87.50)	21 (67.70)
Another race category	1 (1.60)	0 (0.00)	1 (3.20)
Prefer not to answer	1 (1.60)	0 (0.00)	1 (3.20)
Country			
Canada	43 (67.2)	24 (75.00)	30 (96.80)
Ontario	11 (17.20)	11 (34.40)	14 (45.20)
Alberta	7 (10.90)	6 (18.80)	10 (32.30)
Nova Scotia	13 (20.30)	3 (9.40)	2 (6.50)
British Columbia	5 (7.80)	3 (9.40)	1 (3.20)
Quebec	3 (4.70)	1 (3.10)	1 (3.20)
Saskatchewan	3 (4.70)	0 (0.00)	0 (0.00)
Prince Edward Island	1 (1.60)	0 (0.00)	1 (3.20)
Manitoba	0 (0.00)	0 (0.00)	1 (3.20)
United States	13 (20.30)	2 (6.30)	1 (3.20)
Australia	4 (6.30)	5 (15.60)	0 (0.00)
United Kingdom	1 (1.60)	1 (3.10)	0 (0.00)
The Netherlands	1 (1.60)	0 (0.00)	0 (0.00)
Iran	1 (1.60)	0 (0.00)	0 (0.00)
Prefer not to answer	1 (1.60)	0 (0.00)	0 (0.00)
Age (years)			
18–29 years	1 (1.60)	5 (15.60)	16 (51.60)
30–39 years	3 (4.70)	15 (46.90)	5 (16.10)
40–49 years	15 (23.40)	6 (18.80)	5 (16.10)
50–59 years	14 (21.90)	4 (12.50)	5 (16.10)
60 years or greater	8 (12.50)	2 (6.30)	5 (16.10)
Prefer not to answer	1 (1.60)	0 (0.00)	0 (0.00)
Highest level of education			
High school diploma	0 (0.00)	0 (0.00)	1 (3.20)
Some college/professional school	1 (1.60)	0 (0.00)	1 (3.20)
College/professional school diploma	9 (14.10)	0 (0.00)	2 (6.50)
Some undergraduate studies	0 (0.00)	1 (3.10)	4 (12.90)
Undergraduate degree	0 (0.00)	1 (3.10)	12 (38.70)
Some postgraduate studies	3 (4.70)	0 (0.00)	0 (0.00)
Postgraduate degree	17 (26.60)	1 (3.10)	2 (6.50)
Some master’s studies	2 (3.10)	2 (6.30)	2 (6.50)
Master’s degree	12 (18.80)	0 (0.00)	4 (12.90)
Some PhD studies	3 (4.70)	4 (12.50)	1 (3.20)
PhD degree	16 (25.00)	23 (71.90)	2 (6.50)
Prefer not to answer	1 (1.60)	0 (0.00)	0 (0.00)

*N* = 127

**Table 3:** Partner characteristics and KM experience

	Partner group		
	Health professionals	Researchers	Patient/caregiver partners
	*n* (%)	*n* (%)	*n* (%)
Area of KM experience			
Chronic pain	42 (65.60)	18 (56.30)	18 (58.10)
Procedure pain	42 (65.60)	19 (59.40)	11 (35.50)
Acute pain	41 (64.10)	16 (50.00)	6 (19.40)
Rheumatic diseases	22 (34.40)	6 (18.80)	6 (19.40)
Oncological conditions	25 (39.10)	4 (12.50)	6 (19.40)
Haematological conditions	26 (40.60)	3 (9.40)	0 (0.00)
Musculoskeletal conditions	30 (46.90)	7 (21.90)	2 (6.50)
Genetic disorders	25 (39.10)	5 (15.60)	6 (19.40)
Rare disease	11 (17.20)	4 (12.50)	5 (16.10)
Medical complexity	28 (43.80)	5 (15.60)	8 (25.80)
Neurodevelopmental disorders	31 (48.40)	7 (21.90)	11 (35.50)
Another illness/disease	14 (21.90)	2 (6.30)	6 (19.40)
Type of KM experience			
Implementation	60 (93.80)	17 (53.10)	26 (83.90)
Clinical practice change	55 (85.90)	15 (46.90)	15 (48.40)
Policy change	34 (53.10)	5 (15.60)	10 (32.30)
Advisory committee	35 (54.70)	11 (34.40)	20 (64.50)
Evidence adoption/health service improvement plan	34 (53.10)	11 (34.40)	6 (19.40)
Structures for evidence adoption	18 (28.10)	7 (21.90)	1 (3.20)
Decision aids	17 (26.60)	4 (12.50)	1 (3.20)
Local opinion leader	28 (43.80)	5 (15.60)	8 (25.80)
Knowledge broker	12 (18.80)	3 (9.40)	5 (16.10)
Education/training for health professionals	56 (87.50)	12 (37.50)	14 (45.20)
Dissemination	55 (85.90)	32 (100.00)	24 (77.40)
Plain language summary	33 (51.60)	25 (78.10)	13 (41.90)
Policy brief	20 (31.30)	9 (28.10)	8 (25.80)
Materials (e.g. toolkit, patient resources)	48 (75.00)	24 (75.00)	20 (64.50)
Infographics	20 (31.30)	19 (59.40)	14 (45.20)
Arts-based KM	11 (17.20)	5 (15.60)	5 (16.10)
Clinical practice guideline	35 (54.70)	9 (28.10)	3 (9.70)
Position paper	14 (21.90)	4 (12.50)	4 (12.90)
Engaging with knowledge broker	16 (25.00)	6 (18.80)	9 (29.00)
Developing partner network	18 (28.10)	10 (31.30)	6 (19.40)
Social media outreach	16 (25.00)	19 (59.40)	17 (54.80)
Another dissemination activity	5 (7.80)	2 (6.30)	1 (3.20)
KM roles held			
Implementation consultant	31 (48.40)	5 (15.60)	9 (29.00)
Project collaborator	44 (68.80)	20 (62.50)	21 (67.70)
Project leader	35 (54.70)	24 (75.00)	5 (16.10)
Decision maker	20 (31.30)	8 (25.00)	5 (16.10)
Staff carrying out KM	49 (76.60)	10 (31.30)	8 (25.80)
Knowledge user	34 (53.10)	5 (15.60)	20 (64.50)
Another role	0 (0.00)	1 (3.10)	2 (6.50)
Partner types collaborated with			
Researchers	39 (60.90)	29 (90.60)	26 (83.90)
Health professionals	64 (100.00)	29 (90.60)	26 (83.90)
Patients/caregivers	52 (81.30)	29 (90.60)	27 (87.10)
Local partners	43 (67.20)	17 (53.10)	21 (67.70)
National partners	32 (50.00)	15 (46.90)	17 (54.80)
International partners	21 (32.80)	16 (50.00)	10 (32.30)
Level of KM experience			
Expert	28 (43.80)	9 (28.10)	2 (6.50)
Advanced	18 (28.10)	11 (34.40)	12 (38.70)
Competent	16 (25.00)	10 (31.30)	15 (48.40)
Novice	2 (3.10)	2 (6.30)	2 (6.50)
Health profession			
Nurse/nurse practitioner/advanced practice nurse	20 (31.30)		
Psychologist	12 (18.80)		
Physician	10 (15.60)		
Child life specialist	6 (9.40)		
Occupational therapist	6 (9.40)		
Physiotherapist/physical therapist	6 (9.40)		
Social worker	1 (1.60)		
Another health profession	3 (4.70)		
Researcher career stage			
Trainee		11 (34.40)	
Early career		10 (31.30)	
Mid-career		6 (18.80)	
Senior		5 (15.60)	
Area of study			
Medicine/health sciences		13 (40.60)	
Psychology		12 (37.50)	
Nursing		4 (12.50)	
Neuroscience		3 (9.40)	
Physiotherapy/physical therapy		2 (6.30)	
Implementation science		2 (6.30)	
Another area of study		1 (3.10)	
Type of lived experience			
Patient/youth			18 (58.10)
Parent/caregiver			14 (45.20)
Other family member			5 (16.10)
Another type of lived experience			2 (6.50)

KM = knowledge mobilization; health professional *n* = 64; researcher *n* = 32; patient/caregiver partner *n* = 31

### Best–worst scaling results

#### BWS rankings within partner groups

The overall frequencies (i.e. the number of times an item was selected as most and least important) as well as the difference score (i.e. the difference between the most and least frequencies) were calculated for each partner group and represented in bar graphs. Positive difference scores indicate greater importance of an item, whereas negative difference scores indicate lesser importance of an item.

##### Health professionals

**Fig. 4:**
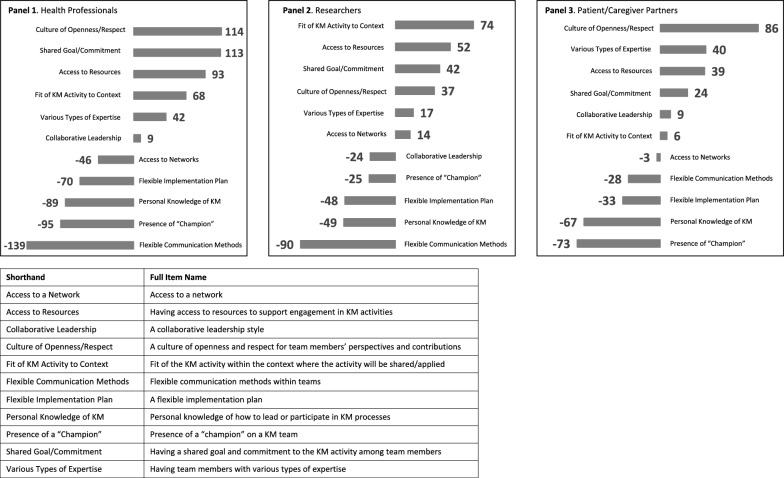
Difference scores of item ratings

The overall frequencies and the difference score were calculated for health professionals (Table 4; Fig. 4, Panel 1). Among health professionals, the three most important items to support their engagement in KM within paediatric pain were “a culture of openness”, “having a shared goal and commitment to the KM activity among team members” and “having access to resources to support engagement in KM activities”. The three least important items were “flexible communication methods within teams”, “presence of a ‘champion’ on a KM team” and “personal knowledge of how to lead or participate in KM processes”. The rescaled values indicated that “flexible communication methods within teams” were less important relative to the other items ranked as least important by health professionals (Table 4).

**Table 4:** Health professionals best–worst scaling total counts and scale calculations

Item	Total counts			Scale calculations		
	Most important	Least important	M − L	sqrt (M/L)	ln (sqrt)	Rescaled
A culture of openness and respect for team members’ perspectives and contributions	123	9	114	3.70	1.31	1.00
Having a shared goal and commitment to the KM activity among team members	125	12	113	3.23	1.17	0.96
Having access to resources to support engagement in KM activities	117	24	93	2.21	0.79	0.83
Fit of the KM activity within the context where the activity will be shared/applied	94	26	68	1.90	0.64	0.79
Having team members with various types of expertise	86	44	42	1.40	0.34	0.69
A collaborative leadership style	48	39	9	1.11	0.10	0.61
Access to a network (e.g. patient/caregiver partner, professional, etc.)	27	73	−46	0.61	−0.50	0.42
A flexible implementation plan	14	84	−70	0.41	−0.90	0.29
Personal knowledge of how to lead or participate in KM processes	28	117	−89	0.49	−0.71	0.35
Presence of a “champion” on a KM team	38	133	−95	0.53	−0.63	0.38
Flexible communication methods within teams	4	143	−139	0.17	−1.79	0.00

Most important = frequency item was selected as most important; Least important = frequency with which item was selected as least important; M-L = most important frequency minus least important frequency; sqrt = square root; M/L = most important frequency divided by the least important frequency; ln = natural log

##### Researchers

Researchers’ three most important items to support engagement in KM within paediatric pain were “fit of the KM activity within the context where the activity will be shared/applied”, “having access to resources to support engagement in KM activities” and “having a shared goal and commitment to the KM activity among team members” (Table 5; Fig. 4, Panel 2). The three least important items were “flexible communication methods within teams”, “personal knowledge of how to lead or participate in KM processes” and “a flexible implementation plan”. The rescaled values indicated that “flexible communication methods within teams” and “a flexible implementation plan” were relatively less important than the other items endorsed as least important (Table 5).

**Table 5:** Researcher best–worst scaling total counts and scale calculations

Item	Total counts			Scale calculations		
	Most important	Least important	M − L	sqrt (M/L)	ln (sqrt)	Rescaled
Fit of the KM activity within the context where the activity will be shared/applied	79.10	5.10	74	3.94	1.37	1.00
Having a shared goal and commitment to the KM activity among team members	48.10	6.10	42	2.81	1.03	0.93
A culture of openness and respect for team members’ perspectives and contributions	43.10	6.10	37	2.66	0.98	0.92
Having access to resources to support engagement in KM activities	63.10	11.10	52	2.38	0.87	0.89
Access to a network (e.g. patient/caregiver partner, professional, etc.)	26.10	12.10	14	1.47	0.38	0.79
Having team members with various types of expertise	33.10	16.10	17	1.43	0.36	0.79
Presence of a “champion” on a KM team	30.10	55.10	−25	0.74	−0.30	0.65
A collaborative leadership style	14.10	38.10	−24	0.61	−0.50	0.61
Personal knowledge of how to lead or participate in KM processes	15.10	64.10	−49	0.49	−0.72	0.56
A flexible implementation plan	1.10	49.10	−48	0.15	−1.90	0.31
Flexible communication methods within teams	0.10	90.10	−90	0.03	−3.40	0.00

Most important = frequency item was selected as most important; Least important = frequency with which item was selected as least important; M-L = most important frequency minus least important frequency; sqrt = square root; M/L = most important frequency divided by the least important frequency; ln = natural log

##### Patient/caregiver partners

Patient/caregiver partners indicated that the three most important items for supporting their engagement in KM were “a culture of openness and respect for team members’ perspectives and contributions”, “having team members with various types of expertise” and “having access to resources to support engagement in KM activities” (Table 6; Fig. 4, Panel 3). The three least important items were “presence of a ‘champion’ on a team”, “personal knowledge of how to lead or participate in KM processes” and “a flexible implementation plan”. The rescaled values indicated that “a culture of openness and respect for team members’ perspectives and contributions” was more important relative to all other items ranked as most important (Table 6).

**Table 6:** Patient/caregiver partner best–worst scaling total counts and scale calculations

Item	Total counts			Scale calculations		
	Most important	Least important	M − L	sqrt (M/L)	ln (sqrt)	Rescaled
A culture of openness and respect for team members’ perspectives and contributions	88	2	86	6.63	1.89	1.00
Having access to resources to support engagement in KM activities	51	12	39	2.06	0.72	0.64
Having team members with various types of expertise	54	14	40	1.96	0.67	0.62
Having a shared goal and commitment to the KM activity among team members	37	13	24	1.69	0.52	0.58
A collaborative leadership style	25	16	9	1.25	0.22	0.48
Fit of the KM activity within the context where the activity will be shared/applied	28	22	6	1.13	0.12	0.45
Access to a network (e.g. patient/caregiver partner, professional, etc.)	19	22	−3	0.93	−0.07	0.39
Flexible communication methods within teams	15	43	−28	0.59	−0.53	0.25
A flexible implementation plan	12	45	−33	0.52	−0.66	0.21
Presence of a “champion” on a KM team	7	80	−73	0.30	−1.22	0.04
Personal knowledge of how to lead or participate in KM processes	5	72	−67	0.26	−1.33	0.00

Most important = frequency item was selected as most important; Least important = frequency with which item was selected as least important; M-L = most important frequency minus least important frequency; sqrt = square root; M/L = most important frequency divided by the least important frequency; ln = natural log

#### Comparison of BWS rescaled scores across partner groups

**Fig. 5:**
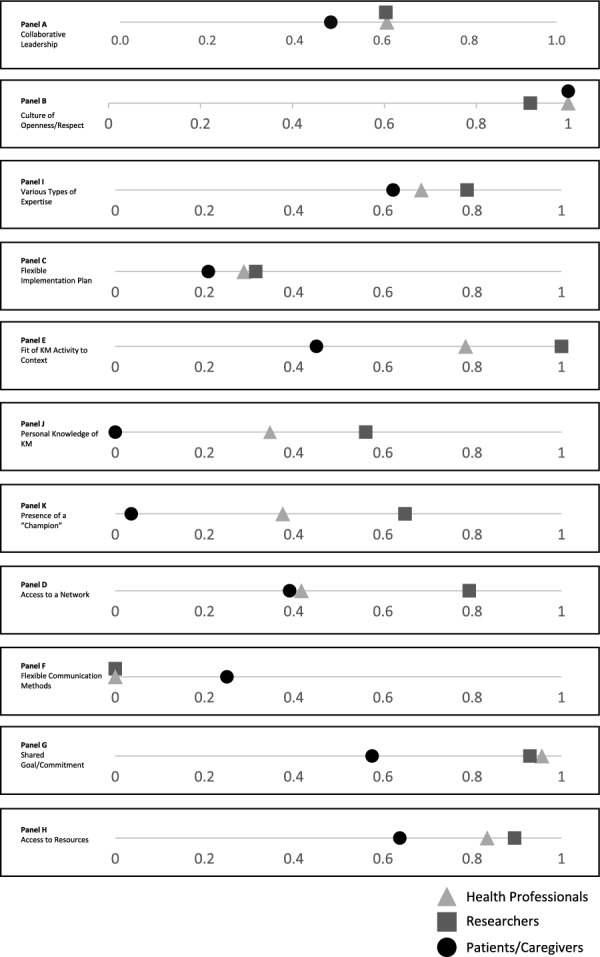
Group-based comparison of rescaled scores by item

The rescaled values presented in Tables 4 through 6 were interpreted to facilitate comparisons in item rankings and importance across the partner groups (see Fig. 5; Panels A–K assigned to each item). To identify perceived differences, panels were visually inspected for ratio scores that did not cluster with (i.e. were not proximal to) those of another partner group. Upon review of all ratio scores that appeared visually distinct from those that were clustered, items with a difference of 0.2 or greater were determined to be relatively different. Four items were determined to have relatively similar importance across groups: “a collaborative leadership style”, “a culture of openness” and “having team members with various types of expertise”, all of which were generally ranked as more important, and “a flexible implementation plan”, which was generally ranked as less important (Fig. 5, Panels A–D, respectively). Three items were determined to be relatively different across groups: “fit of the KM activity within the context where the activity will be shared/applied”, “personal knowledge of how to lead or participate in KM processes” and “presence of a champion on a KM team” (Fig. 5, Panels E–G, respectively). Researchers endorsed all of these items as being relatively more important than health professionals, who endorsed these items as being relatively more important than patient/caregiver partners. Finally, there were four items that were similar between two groups but relatively different from the third. The item “access to a network” was relatively more important to researchers than it was to health professionals and patient/caregiver partners (Fig. 5, Panel H). The item “flexible communication methods within teams” was relatively more important to patient/caregiver partners than it was to researchers and health professionals (Fig. 5, Panel I), whereas “having a shared goal and commitment to the KM activity among team members” and “having access to resources to support engagement in KM activities” were relatively less important to patient/caregiver partners compared with the other two groups (Fig. 5, Panels J and K, respectively).

#### Data quality

Among all participants, 81.1% (*n* = 103) indicated that they understood the BWS questions, while 1.6% (*n* = 2) did not and 16.5% (*n* = 21) were unsure. Overall, 68.5% (*n* = 87) reported that they considered all items every time, 29.9% (*n* = 38) reported that they considered all items more than half the time and 0.8% (*n* = 1) reported that they considered all items less than half the time. No participants reported “not ever considering” all the items. One participant did not respond. Review of the duplicate choice sets showed that 42.5% (*n* = 54) of responses were identical between the two items. The rating of one item was different between sets in a total of 44.9% (*n* = 57) responses (e.g. a different “most important” item was selected in the duplicate set than was selected in the initial set). Both items were different in 12.6% (*n* = 16) of responses (i.e. both most and least important items changed in the duplicate set). A potential explanation for this occurrence is that items selected as the most and least important may change as participants review and compare other items. Given the consistency of responding between the original and duplicate items, and the fact that participants predominantly attended to all or most items the overwhelming majority of the time, the data quality is considered acceptable [31, 33].

## Discussion

This study presents an investigation of factors that support the engagement of health professionals, researchers and patient/caregiver partners in KM activities within paediatric pain. It is also the first study to quantify these preferences and priorities using a best–worst scaling experiment. This adds a unique approach within the implementation science literature that adds an experimental perspective on the understanding of how knowledge users and producers prefer to be engaged in partnerships. This approach specifically aids in developing the understanding of decisional considerations and mechanisms that underlie partnership in KM activities. The results of this study indicated that when engaging in KM partnerships, partner groups share many priorities. Items consistently ranked as more important across the groups were “a culture of openness and respect for team members’ perspectives and contributions”, “having a shared goal and commitment to the KM activity among team members”, “having team members with various types of expertise” and “having access to resources to support engagement in KM activities”. Items consistently ranked as less important were “a flexible implementation plan”, “personal knowledge of how to lead or participate in KM processes”, “presence of a champion on a KM team” and “flexible communication methods within teams”. Other items differed in their rank as more or less important among groups (e.g. “access to a network”). Patient/caregiver partners’ priorities relatively differed the most from the other two groups, whereas health professionals differed the least. This may suggest that health professionals could act as a unifying presence on KM teams, through their ability to align with other partner types and find solutions that address the needs of multiple partners through their relatively similar priorities.

### Common and unique priorities for supporting partnership in KM

In considering the clusters of items endorsed as most and least important across all groups, two key trends emerge. First, many of the items among the most important across partner groups relate to the concept of team culture or relationship quality among team members (e.g. culture of openness and respect, shared goals and commitment, team members with various types of expertise), while the items more frequently selected as least important are more closely related to the pragmatic elements of KM (e.g. flexible communication methods, flexible implementation plan, knowledge of how to lead or engage in KM). This suggests that relationships and culture within KM teams are more important to establishing and maintaining relationships than practical elements pertaining to how KM and partnerships are carried out. Given the importance of pragmatics as enablers in partnerships [8], this result may seem contrary; however, consideration of the role of relationships within teams and projects in the broader literature aligns well with these results. For example, in the health services and psychology literature, positive team dynamics and cohesion foster bonding, motivation and commitment among team members to work towards a shared goal [39–41]. These positive interpersonal factors create potential for greater coordination, collaboration and team efficacy [39–41]. In addition, a sense of positive affect within the team is related to greater connectedness between, and integration among, team members, which fosters a sense of psychological safety within teams [39, 40, 42]. For example, a model proposed by Knight and Eisenkraft indicated that positive and negative affect within groups is respectively associated with strong or weak social integration, which in turn is associated with improved or worsened task performance [42]. That is, when teams have positive regard, they are more likely to feel integrated with their team members, which increases the likelihood that task outcomes will be favourable.

Trust among team members has also emerged as a key concept within participatory health research contexts, with a key opportunity for the development of trust being in the initiation and organization of a research network itself, as well as how partners engaged with each other, in terms of frequency and quality of interaction [16, 39, 43]. Concepts around trust and belongingness arose in the qualitative study preceding this work, which suggests that it may underpin the present findings as well [18]. Thus, it may be that positive relationships promote better coordination of practical elements of partnerships when there is positive affect and trust at the foundation of the partnership. This may be particularly important to account for in the context of paediatric pain, given that a lack of trust between patients and health professionals can influence the success of their clinical relationship [44]. Investigations into the role and nature of relationships, and team culture within KM specifically, have been relatively limited [45]; however, the results of this study indicate that what is known about relationships and partnerships in health contexts more broadly may be applied in the context of KM. Interpersonal elements of KM are context dependent and may differ from team to team [27, 46] and thus may be less easily modified relative to more so-called practical constructs such as communication methods or gaining knowledge on KM processes. Regardless, these interpersonal factors are evidently equally if not more important to further understand in the context of partnership and implementation science. Thus, future research should continue to examine the applicability of existing frameworks or partnership approaches within the research context that account for interpersonal and team dynamics in the KM partnership context. The modification and application of these existing approaches when initiating and maintaining partnerships could promote well-integrated and effective KM teams.

The second key finding was that while these overall trends were apparent across groups, there were specific priorities that emerged within each individual group. For example, higher priorities among researchers were the “fit of the KM activity within the context where the activity will be shared/applied” and “access to a network” relative to other partner types, whereas patients/caregivers prioritized “flexible communication” as more important relative to the other partner types. Furthermore, patient/caregiver partners ranked many items as being relatively less important than the other partner groups, such as “fit of the KM activity within the context where the activity will be shared/applied” and “personal knowledge of how to lead or participate in KM processes”. Thus, while items were relatively consistent across groups in terms of whether they were ranked as most or least important, the differences in item rankings within these categories indicates nuance in how these overall values of relationships or pragmatics may be addressed for each partner type. This was especially apparent among patient/caregiver partners who had the most item ranking differences compared with the other two groups. In general, the differences in item rankings speak to the importance of tailoring approaches to partnership, especially when different partners have unique knowledge and interests that support the KM initiative. Within KM, tailoring is a key approach to ensure the evidence being disseminated is relevant to the needs of the audience, and adapted in such a way that it will be easily applied in practice, ultimately improving the uptake of evidence [47, 48]. This is especially critical to inform practice for KM within paediatric pain, where it is clear that distinct approaches are necessary to support the implementation of evidence in practice [1]. By taking this tailored approach, individual partners may be better able to share relevant perspectives on what aspects of pain and related evidence should be mobilized. While there is very limited evidence available to inform how tailoring applies to KM partnerships, the broader implementation literature describes tailoring as giving consideration to the implementation context and selecting specific strategies that address the needs of the individuals [49–51]. Further research is needed to understand how approaches to tailoring, such as concept and intervention mapping (i.e. processes that inform how interventions are developed and implemented), may be applicable to the partnership context specifically [51, 52]. Even within the broader implementation science literature, approaches to selecting and tailoring implementation strategies are infrequently informed by evidence or the context in which they are to be used, thus limiting their relevance and impact [53]. Thus, there is a clear need to better understand how to tailor approaches to partnership and implementation more broadly. Future research should endeavour to explore strategies to tailor approaches to, and strategies for, partnerships.

### The need for a structured approach to partnership

The evidence that there are relative differences in how groups ranked individual items serves as a reminder that the manner in which relationships and pragmatics are addressed also cannot be uniform; that is, a one size fits all approach to addressing these concepts is not appropriate. Even the way individual factors are experienced or implemented differs among partner groups [18]. This type of nuance must be accounted for when establishing and evaluating partnerships to ensure priorities are addressed appropriately. Use of empirically informed tools may support this approach. In general, there are limited validated approaches to evaluate the impact of patient engagement in research [54, 55]; however, tools such as the Patient Engagement in Research Scale (PEIRS) have filled this gap by providing an approach to evaluating the quality of partnerships in research [56] and may be appropriate to inform such assessments or evaluations. The present study results suggest that components of the PEIRS, such as feeling valued and quality of team interactions, support and contributions, are factors that bear relevance to the KM context. Further validation of the relevance of this approach in the context of KM partnerships is necessary, as is the addition of factors that pertain to relationship development, especially with partners who are not patients (e.g. researcher, health professionals). Moreover, while this tool may lend itself well to partnership evaluation, there remains a lack of structured tools available to assess these preferences and priorities at partnership initiation. Other tools such as the Research Quality Plus for Co-Production (RQ + 4 Co-Pro) or the Patient Engagement Evaluation Tool (PEET), which can inform and assess the quality of partnerships between knowledge producers and users in evidence co-production partnerships, may also be used to inform and evaluate how partnerships are developed [57–59]. While these tools are not specifically designed primarily with partnership for KM activities in mind, they may provide indicators to support these types of activities (e.g. contextual factors, legitimacy). Overall, helpful and relevant tools have been developed in partnership for evidence co-production, yet further work is needed to adapt existing tools for this purpose or create specific tools for the KM partnership context.

## Strengths and limitations

This study had a number of strengths that enriched the quality of the results and their generalizability to partners engaging in KM. First, the use of experimental methods is a significant strength of this approach. BWS is a unique experimental approach that not only facilitates developing an understanding of what factors individuals value when making decisions but also ensures that the factors presented are ecologically valid and modifiable in the contexts where they will be used. In the context of the present work, this ensures that the findings of the experiments are inherently transferable to practical settings. From a data quality perspective, the use of BWS as the experimental method offers several advantages over traditional ranking or rating scales. BWS operates on the assumption put forth by the adaptation level theory that humans are more reliable in their responding when asked to select extremes [36, 60]. Furthermore, when participants provide information about items at extremes of preference or importance, this provides more information than asking about a most preferred option alone [36]. Another significant strength of this study was the rigorous process through which items were generated, using in-depth qualitative data from partners with KM experience [18]. This approach ensured the appropriateness of the items and their definitions, dimensions and context [61]. Partnership and collaboration with the expert panel was also key to the development and data interpretation, with feedback from the cognitive interviews not only informing the development of the study design and materials but also the lens through which the results were interpreted. This was paramount to the success of this study and its potential impact by ensuring the integrity and relevance of the results. The strength of the broader partnership-based approach will also be leveraged in disseminating these results to key partners who engage in KM activities within paediatric pain through relevant pain organizations and networks.

This study was not without its limitations. While the BWS experiment offered many strengths in terms of its ability to understand how people prioritize various factors in the context of KM partnership, the BWS design itself does not facilitate an understanding of what participants think about the choice itself (i.e. whether discussing priorities when engaging in KM partnerships is relevant in the first place). The empirical data with which this study was developed [18], along with the review of the expert panel, was used to avoid this issue; however, it is possible that this question did not bear relevance to every participant. Future BWS research studies may consider asking about relevance. Another limitation is the potential for sampling bias. The recruitment methods chosen made it challenging to calculate a response rate from these sources for a variety of reasons (e.g. listservs and networks did not have exact membership counts). Therefore, the extent and impact of sampling bias in the present study is unclear. Regarding the results, a limitation is that some of the item ranks changed slightly depending on whether the difference scores or ratio values were used. This phenomenon has been anecdotally observed in other published papers (e.g. Louviere and Flynn) [62]. Despite the slight differences in item rankings across the two values, the categorization of the items as most or least important did not change. Future research into this phenomenon is needed to confirm why this difference occurs. Finally, this sample was predominantly composed of white women. The experiences of individuals who engage in KM are known to be influenced by race, ethnicity and gender [63–66]; therefore, these results are limited in their generalizability to individuals from equity-deserving groups and to KM partners of other genders. Given the unique interpersonal and systemic challenges that may be faced by these groups, specific investigations into nuances that may exist in terms of priorities in partnerships are warranted.

## Conclusions

Partnerships within KM are essential to ensure the best available evidence is effectively integrated into practice and policy. As partnerships between various partner types continue to become the norm in KM approaches, evidence-informed and concerted efforts are necessary to ensure partners are engaged in the most effective and appropriate way possible. Partners share many similar priorities in terms of how they wish to approach partnerships, with relationship quality being an important priority; however, there is also a great deal of nuance in terms of the unique ways in which different types of partners wish to have those relationships and other partnership priorities addressed. The opportunity to account for these nuances and priorities can promote more effective KM activities for paediatric pain. In turn, the more effective uptake of what are ultimately more relevant and targeted initiatives holds great promise for translating into more effective pain management for children. Thus, individuals leading KM initiatives within paediatric pain and children’s health should approach partnership with curiosity and sensitivity to how partners wish to be engaged to ensure partnerships operate as successfully as possible.

## Supplementary Information

Additional File 1.

## Data Availability

The datasets used and/or analysed during the current study are available from the corresponding author on request.

## Abbreviations

BIBD: Balanced incomplete block design
BWS: Best–worst scaling
CAD: Canadian dollar
CFIR: Consolidated Framework for Implementation Research
KM: Knowledge mobilization
PEIRS: Patient Engagement in Research Scale
REB: Research Ethics Board
SD: Standard deviation
STROBE: Strengthening the Reporting of Observational Studies in Epidemiology
